# The Metropolitan Medical Schools

**Published:** 1896-09-05

**Authors:** 


### The Metropolitan Medical Schools.

### MEDICAL STUDY IN LONDON.

When the would-be student has decided to adopt medicine as a profession, the choice of a medical school in which to obtain the necessary education presents considerable difficulties, especially to those who are not led by their circumstances, by their place of residence, by friendship with men who are already students, or by their desire to follow in the footsteps of their father or other relative, to attach themselves to any particular hospital or school of medicine.

Individual desires and idiosyncrasies will turn different men to different centres of education. Some will prefer London, some the provinces, some Scotland, others Ireland; and among each group there will be those who will choose, or who, by circumstances, will be driven to this, that, or the other school of medicine. There can, however, be but little doubt that to the man who is free and unfettered in his choice, and is ready and willing to take trouble and to lay out his time to the best advantage, London offers a field for study such as is possessed by few other places in the world.

Among the medical schools of London, however, there is sufficient variety to cause no small embarrassment, and in truth there is no one medical school which has so exclusive a monopoly of all the virtues that one can say without hesitation—that is the best school.

No doubt, for those who take the view that life is short, and that the brain at the best is only capable of absorbing a certain amount of knowledge in the time relegated to study, there will be apparent advantage in attaching themselves to schools such, for example, as certain of the Scotch Universities, in which the art of teaching has been profoundly studied, and where the daily course which shall eventuate in an M.D. is laid down with great exactitude. But, for broader-minded men, we have no doubt of the advantages of London, or that, if once the University were brought in touch with the wants of the medical profession, so that a degree in medicine could be attained with the same facility here as it is in Scotland, London should stand head and shoulders above all other places as a centre for medical study for English-speaking people. There is no medical school in London which has not on its staff men who are well known—nay, celebrated—in their own departments, and men who are thoroughly well competent as teachers. On the other hand, there is probably no school in London which has not one or more "duffers" in its teaching staff; men who, however deep their knowledge, have failed to learn the art of conveying it to others. There is, then, no one school which is all good, any more than there is any one which is all bad, or even indifferent. The variety between the different schools cannot be expressed in such terms. One is strong on one side, one on another. One has a reputation for the clinical teaching in its wards, another for the systematic teaching in its class-rooms. One may have the best dissecting-room, another the best laboratory, and so on. Then some schools are more expensive than others, and it is probably very often true that the more expensive schools are frequented by students who, having more money than others, have more expensive tastes, and thus it may well occur that the general tone, the average standard of living, of dress, and of amusements, at one school may be of a more expensive type than at another; and although this is an influence from which a student with any backbone may, if he chooses, shake himself free, it is a matter not to be entirely ignored in the choice of a place of study for a youth of a sensitive disposition, but of a slender purse. On the other hand, it must not be forgotten that some schools are much better provided with prizes and with scholarships than others are, and although we would much deprecate the plan of arranging a student's finances on the very slender basis of his being able materially to assist himself by gaining scholarships, still, the extent of the provision of such aids to comfortable subsistence offered by the different colleges is a point to be carefully considered by those whose brains are of that peculiar type which enables their owners to glide easily through the strange pitfalls of the examination-room. We hesitate to say that the prizes are to the strong, or that the student who, term after term, is decorated with medals and carries off arms full of prizes is of necessity the best man or will make the best practitioner; but certainly some men and boys have the knack of so formulating and presenting their knowledge as to pass examinations with comparative ease, and to such the number and value of scholarships at a given school is a matter of no small importance, while to the lad who after all his schooling can only just scrape through his entrance examination such prizes are a matter of indifference, they are too far beyond him for consideration. The question then is not, which is the best medical school in London? but, which is the most suitable in the particular case? For the comfort, however, of parents and guardians, and for the stimulation and encouragement of students, who are sadly apt, so soon as they are fixed at one place, to raise loud lamentations that they had not been sent to somewhere else, we would say that we are quite certain that at the present time there is no medical school in London at which an intelligent and careful student may not qualify himself to pass his examination with credit and to practise his profession with distinction.

Not including the London School of Medicine for Women, there are in London eleven hospitals which have medical schools. These eleven schools may be conveniently divided into three classes. There are, in the first place, the three great endowed hospitals, St. Bartholomew's, Guy's, and St. Thomas's, each of which has its school attached.

The hospital of St. Bartholomew was founded in 1123 A.D., and there are records of a medical school being connected with the hospital as early as 1662. The hospital contains 744 beds, and is a wealthy institution. Needless to say that St. Bartholomew's is one of the largest of the London schools, and has a proportionately large staff of teachers.

Guy's, again, is one of the great London hospitals, and one of the largest of the medical schools. It is thoroughly well equipped for teaching, and, in consequence of the great pecuniary assistance which the hospital has received this year, the clinical opportunities are likely to be largely increased.

St. Thomas's, occupying a fine position opposite the Houses of Parliament, is structurally one of the finest hospitals in the world; the school is a thoroughly good one, and students with such advantages ought to do well.

Then there are in London two completely equipped colleges—King's College and University College. Each of these has professors in many branches of knowledge, science, art, language, history, engineering, &c., as well as a complete medical department, and attached to each is a hospital for clinical teaching. Theoretically, these colleges ought to offer many advantages over mere schools connected with hospitals. One might expect that with their large number of students and their wide variety of studies, their museums and libraries concerned with a multitude of interests outside the field of medicine pure and simple, and with the commingling of students with many aims and varied ambitions, an education of much wider scope, a culture of much broader sympathies might be attainable than at schools where all are engaged in the same course of study, all running along the same narrow track which, without side issues or collateral interests, is laid down as the shortest route by which a diploma may be reached. We fear that in practice this is not so, and that at the colleges, as at the schools, the medical students keep together and take but little interest in what lies outside the exact curriculum laid down by the Examination Board. It is a pity, and we think that every effort should be made to render these colleges something better than an accidental conglomeration of many class-rooms under one roof.

Then come the six medical schools which neither form part of colleges nor are connected with endowed hospitals. They are attached to the following hospitals: Charing Cross, St. George's, The London, St. Mary's, Middlesex, and the Westminster. These schools are, one may say, in every case the direct outcome of the energy and fame of the medical staffs of the hospitals to which they are attached, and though by accident we mention them last, they are in no way inferior to those that have come before, some of them being second to none in their equipment and their teaching power, and all of the hospitals with which they are connected being full of useful clinical material. Lastly, there is Mr. Cooke's School of Anatomy, Physiology, and Surgery, at which special classes can be taken by students who require extra help for special purposes.

List of London Medical Schools, Giving the Cost of Education at Each.

| Name of Medical School. | Fee for all Lectures and for Medical and Surgical Practice in the Hospitals. | Times of Payment. |
|---|---|---|
| St. Bartholomew's Hospital ... | £157 10 0 | Or first year, £42 instalment fees; second year, £42; third year, £42; fourth year, £42. |
| Charing Cross Hospital ... ... | 110 guineas. | Or first winter, 33 guineas; first summer, second winter, second summer, third winter, 22 guineas each. The sons of registered medical practitioners pay 100 guineas in one sum, or 110 guineas in five instalments. |
| St. George's Hospital ... ... | £145 0 0 | Or first year, £45; second year, £45; third year, £40; fourth year, £20. |
| Guy's Hospital ... ... ... | £157 10 0 | Or first year, £42; second year, £42; third year, £42; fourth year, £42. |
| King's College and Hospital ... | £135 0 0 | Or in two annual instalments of £70, or three of £48, or four of £37 each. |
| London Hospital ... ... ... | 120 guineas | Or 130 guineas by three instalments; first year, 45 guineas; second year, 45 guineas; third year, 40 guineas. A reduction of 15 guineas is made to the sons of members of the profession. |
| St. Mary's Hospital ... ... | Perpetual Studentship, £139 0 0 | Or first year, £46; second year, £40; third year, £30; fourth year, £28. |
| Middlesex Hospital... ... ... | 120 guineas | Or 130 guineas by three instalments. |
| St. Thomas's Hospital ... ... | 150 guineas | Or by two instalments of £85 and £72 10s.; or by three instalments of £75, £50, £32 10s.; or by four instalments of £65, £50, £30, and £12 10s. |
| University College and Hospital | £141 15 0 | Or by instalments of £68 5s., £52 10s., and £26 5s. |
| Westminster Hospital ... ... | 100 guineas | Or two instalments of 55 guineas each; or six instalments of 25 guineas and 15 guineas alternately. |

*** The London School of Medicine for Women is given on page 375.

